# Supplementary material for: Negative DNA supercoiling makes protein-mediated looping deterministic and ergodic within the bacterial doubling time
Source: Nucleic Acids Res. 2021 Nov 1;49(20):11550–9. doi: 10.1093/nar/gkab946 (PMC8599721; doi:10.1093/nar/gkab946)
Supplement: gkab946_Supplemental_File [file gkab946_supplemental_file.pdf]

Figure S1

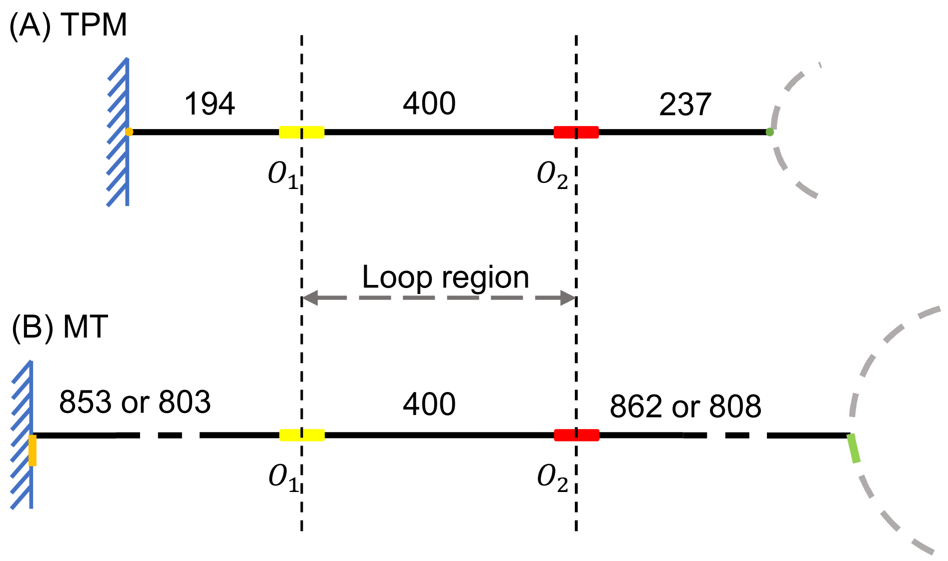

Figure S1. Schematic diagrams of the DNA constructs used in TPM and MT measurements. Both constructs have the same central 400 bp loop region segment flanked by the O1 operator, toward the attachment to the glass surface of the microchamber (left, hatched) and O2 operator, toward the bead (right dashed arc). Lengths are given in base pairs.

Figure S2

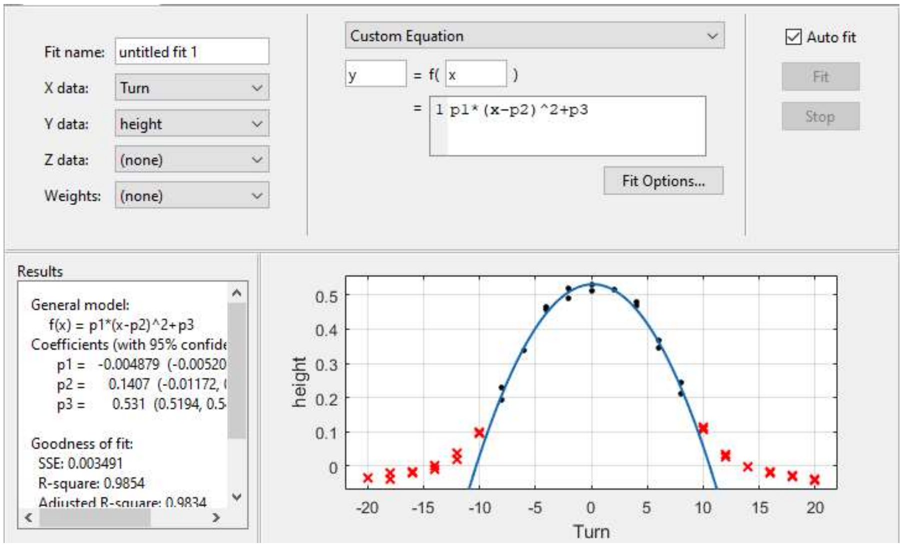

		Turn	CorrectTurn	Supercoil D	Ploop
		-1	-1.1407	-0.5609116	0
Center	0.1407	-2	-2.1407	-1.0526374	0
Height	0.531	-3	-3.1407	-1.5443631	0
		-4	-4.1407	-2.0360889	4.918486
		-5	-5.1407	-2.5278147	42.32796
		-6	-6.1407	-3.0195404	93.48375
		-7	-7.1407	-3.5112662	98.72791

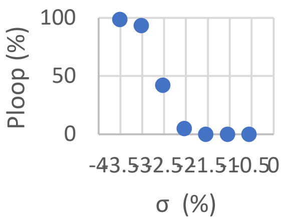

Figure S2. **Finding the center of a twist versus extension curve.** The number of turns added to a DNA molecule was calculated by fitting the extension versus twist curve with a parabola to determine the center.

Figure S3

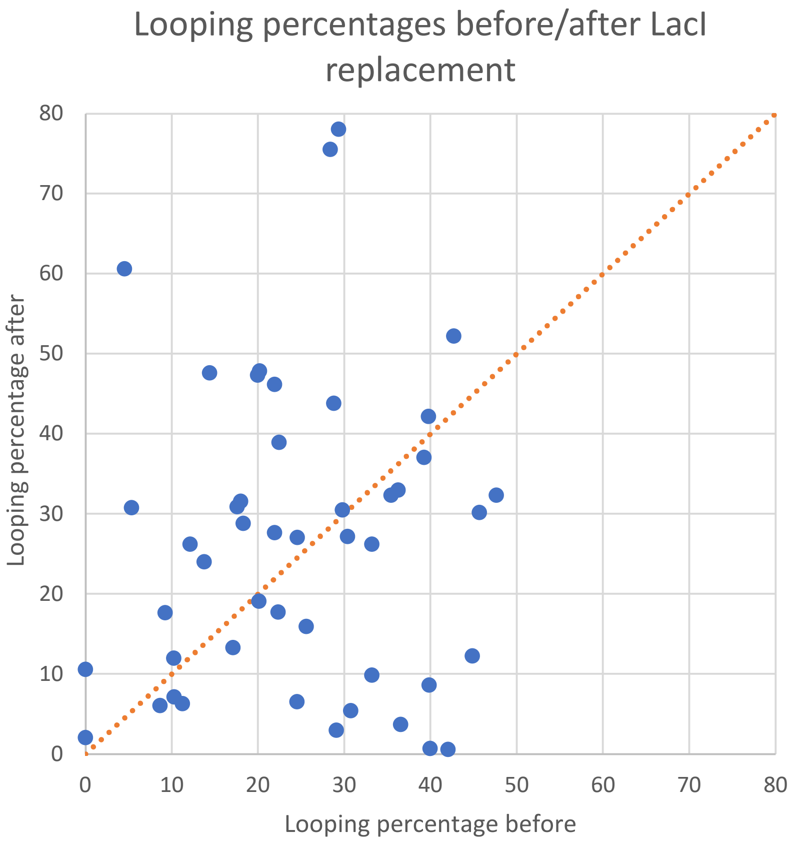

Figure S3. Looping percentages are uncorrelated before and after LacI replacement.
Using tethered particle motion analysis, the percentage of time spent in the looped state was measured for a field of view of individual tethers in a first observation period. Then LacI was washed out and re-introduced. Following that the percentage of time spent in the looped state for the same set of tethers was assessed for a second observation period. The data spread almost evenly above and below the line Y=X (51% vs. 49%). A Pearson's correlation coefficient of 0.066 indicates poor correlation. The mean looping percentages before and after are 25+/-13 and 29+/-19, which are indistinguishable by t-test (0.61).The lack of correlation between looping percentages measured for individual tethers before and after LacI replacement indicates that the percentage of looping is not conditioned by a given DNA molecule.

Figure S4

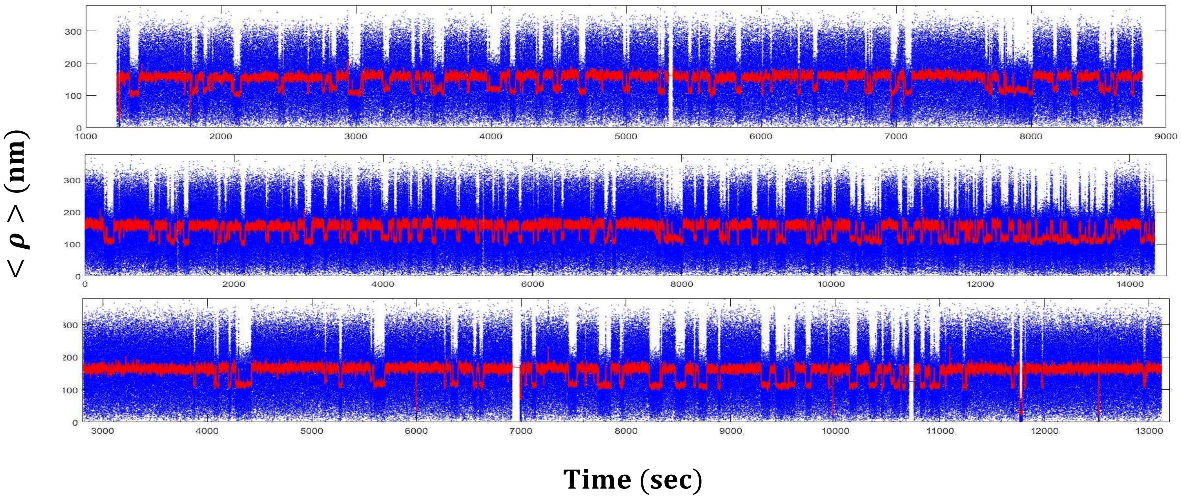

Figure S4. Long TPM recordings. Three representative recordings are shown from a set of fifty lasting over one hour. The blue dots represent the two-dimensionally projected amplitude of momentary excursions of the tethered bead; the red trace is the moving, 8-second time average of these values.

Figure S5

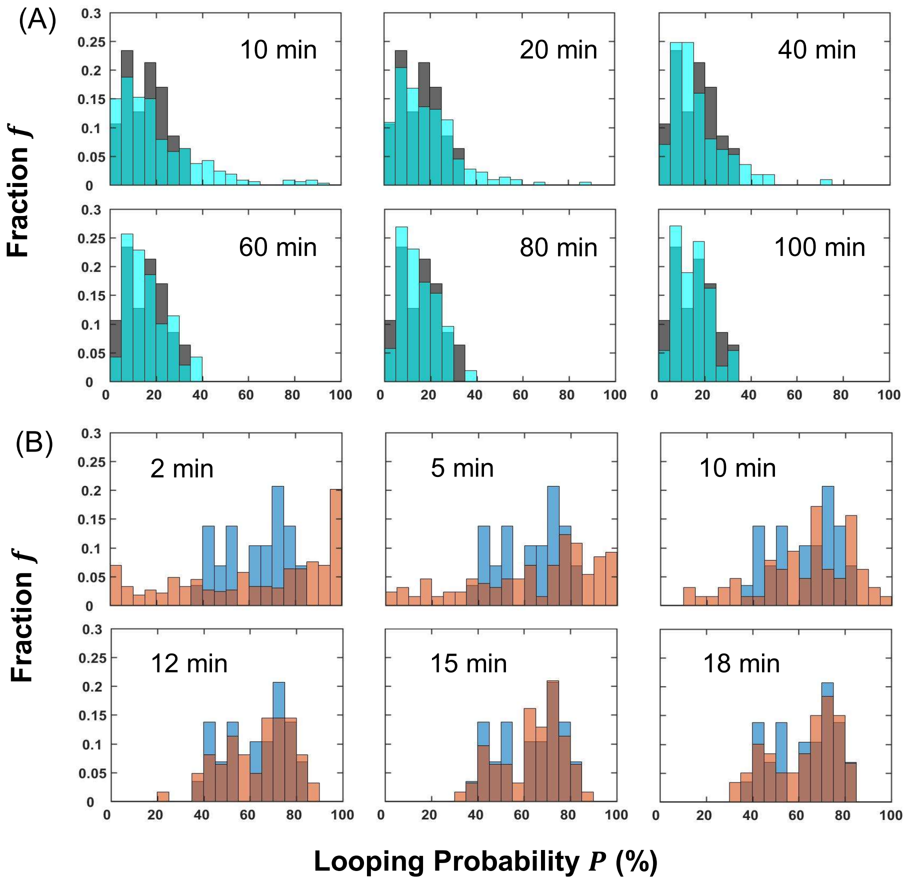

Figure S5. Sufficiently long observations are ergodic.
(A) Distributions of the looping probabilities derived from TPM records lasting 5 hours (black) or split into shorter segments as indicated (cyan). (B) Distributions of the looping probabilities derived from MT records lasting 20 min (blue) or split into shorter segments as indicated (brown). All data was recorded at 0.45 pN of tension and -1.5% supercoiling. The distributions of looping probabilities for the corresponding full-length record is included for comparison in each panel.

Figure S6

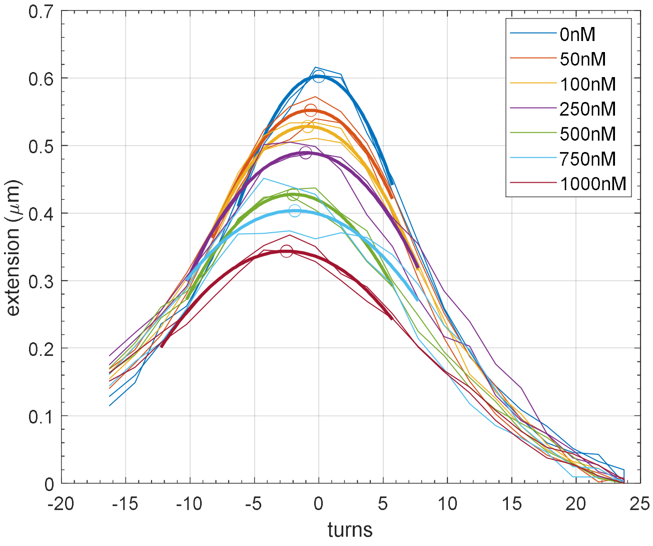

Figure S6. HU binding significantly contracts but mildly unwinds DNA. A single 3352 bp DNA tether under 0.45 pN of tension in a magnetic tweezer was repeatedly wound and unwound in a buffer containing 200 mM KCl with various concentrations of HU ranging from 0 to 1000 nM. Increasing HU concentrations steadily reduced the extension of the DNA tether by nearly 50% but produced only very mild negative supercoiling. A parabolic curve (thick) was fit to each pair of winding and unwinding curves corresponding to different concentrations of HU. The vertex of the parabola represents the shift of the twist versus extension curve due to the supercoiling induced by HU protein binding to the DNA tether.

Figure S7

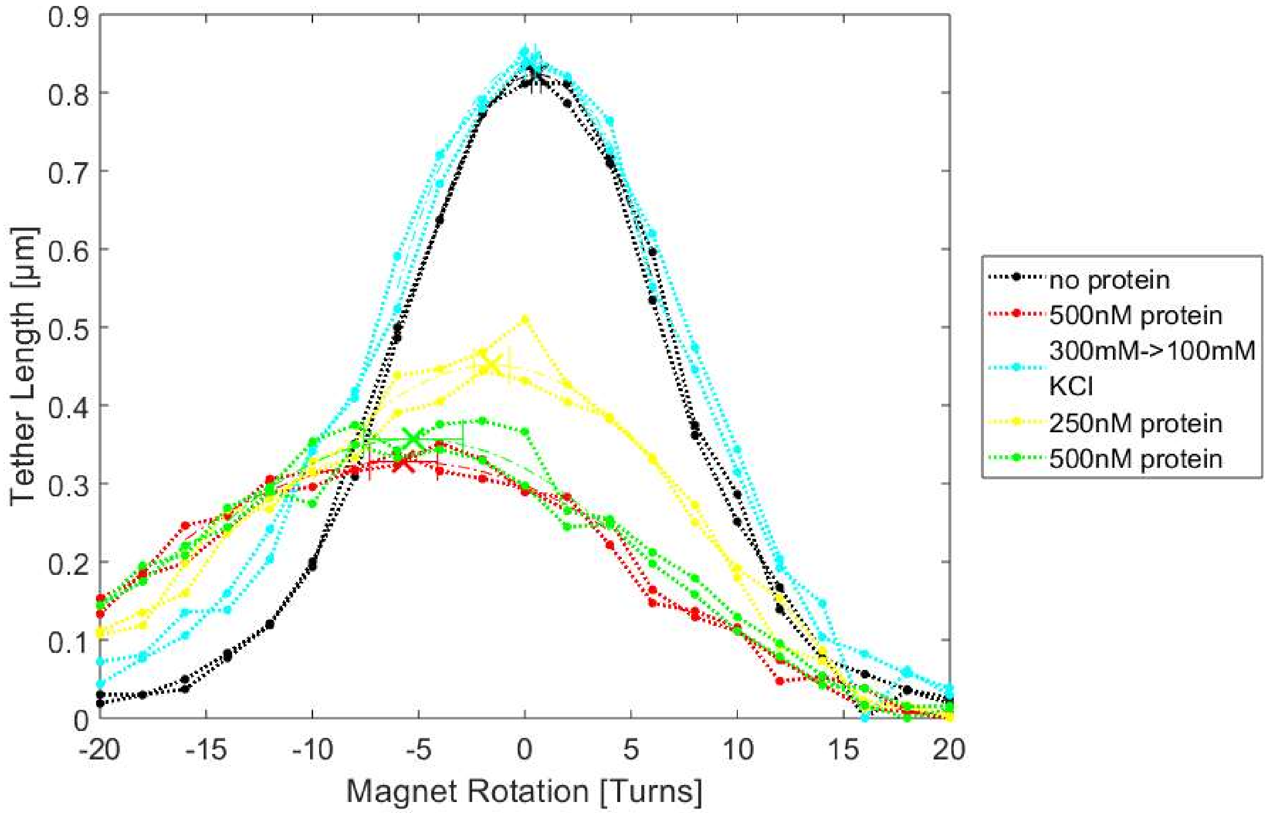

Figure S7. While HU dissociates in high salt , it does not dissociate under a wide range of supercoiling levels. Extension versus twist curves were recorded before introducing 500 nM HU, in 100 mM KCl, after washing away HU with high salt, and after stepwise re-introduction of 250 followed by 500 nM HU. 500 nM protein shifted and reduced the maximum extension of the curve. 300 mM KCl dissociated HU and restored the curve to the original position and height. Introduction of 250 nM HU partially shifted and reduced the maximum extension of the curve. Re-introduction of 500 nM HU shifted and reduced maximum extension of the curve to the levels observed previously.

Figure S8

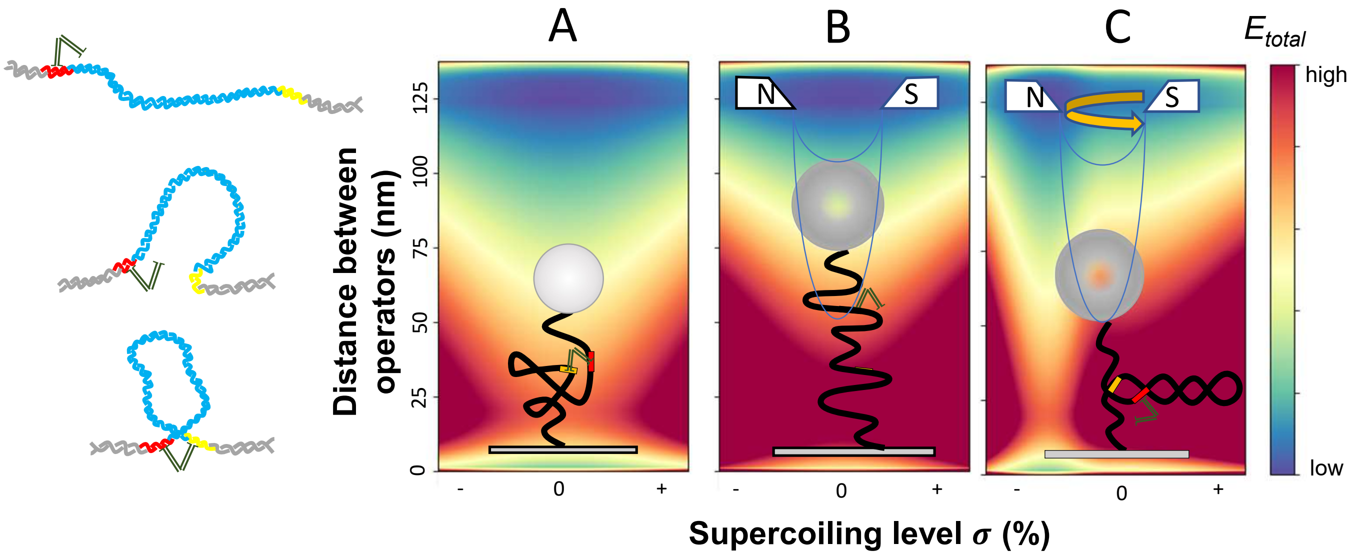

Figure S8. Energy landscapes for loop closure by LacI in different conditions of tension and torsion. The illustrations at the left show possible conformations of DNA tethers corresponding to different separations between the operators. *A-C* are three hypothetical energy landscapes for a DNA tether under different conditions of torsion and tension are represented. The energy values are qualitatively encoded using the color scale at right. The *y*-axis indicates the distance between the protein binding sites that constitute the junction and may vary between zero and 133 nm for a 400 bp DNA segment. DNA supercoiling varies along the *x*-axis. Superimposed on each panel are illustrations of likely DNA conformations under the different conditions of torsion and tension.

Table S1

Construct	Template	Sense primer	Anti-sense primer	Restriction enzymes
O1-400-O2 (TPM)	pO1O2[1]	D-S/pO1O2_401/1588	B-A/pO1O2_401/2418	none
O1-400-O2 (MT)	pO1O2, pZV_21_400[2]	S/pO1O2_401/929_XmaI, S/JBOIDO1_400/1799_Apal	A/pO1O2_401/3043_ApaI, A/JBOIDO1_400/3810_Xmal	XmaI, ApaI
3352 bp segment (MT)	pYY_I1_400_BstEII[3]	S/JBOIDO1_400/1783-Apa1	A/JBOIDO1_400/5113-Xma1	XmaI, ApaI
Bio-, dig-tails	pBluKSP+	A/pIT_Loop3/1665	Pol1979R	XmaI, ApaI

Table S2

Primer name	Sequence
D-S/pO1O2_401/1588	[dig]-tgctcgcttcgctacttg
B-A/pO1O2_401/2418	[bio]-tgactgggttgaaggctc
S/pO1O2_401/929_XmaI	tgCCCGGGacccggaaagacatgc
A/pO1O2_401/3043_ApaI	ctGggCCCggtgaatccgttagcga
S/JBOIDO1_400/1997_ApaI	acccgtGGGCCCagcatcctctctcgtttcatc
A/JBOIDO1_400/3810_XmaI	gcagcgCCCGGGtgagcgaggaagcggaagag
A/pIT_Loop3/1665	ggcgattaagttgggtaacg
Pol1979R	tgtggaattgtgagcggata
A/JBOIDO1_400/5113-Xma1	acaacacccgggATCATGTAACTCGCCTTG
S/JBOIDO1_400/1783-Apa1	atgttcgggcccAGTAACCCGTATCGTGAGC

Supplementary plasmids and primers. Table S1 (Top) The DNA constructs were constructed using plasmid templates with the indicated pairs of primers. **Table S2** (Bottom) Sequences for each primer. Bio- or B- is a biotin tag at the 5’ end of the primer, and D- or dig- is a digoxigenin tag at 5’ end of the primer.

[1] G. Fulcrand *et al.*, DNA supercoiling, a critical signal regulating the basal expression of the lac operon in Escherichia coli. *Scientific reports* **6**, 19243 (2016)

[2] Z. Voros, Y. Yan, D. T. Kovari, L. Finzi, D. Dunlap, Proteins mediating DNA loops effectively block transcription. *Protein Science* **26**, 1427-1438 (2017)

[3] Y. Yan, F. Leng, L. Finzi, D. Dunlap, Protein-mediated looping of DNA under tension requires supercoiling. *Nucleic Acids Res* **46**, 2370-2379 (2018)
